# Effects of partial dietary supplementation of fish meal with soymeal on the stress and apoptosis response in the digestive system of common dentex (*Dentex dentex*)

**DOI:** 10.1186/s40709-017-0071-1

**Published:** 2017-12-21

**Authors:** Efthimia Antonopoulou, Eleni Chouri, Konstantinos Feidantsis, Antigone Lazou, Stavros Chatzifotis

**Affiliations:** 1 Laboratory of Animal Physiology, Department of Zoology, School of Biology, Aristotle University of Thessaloniki, 54124 Thessaloniki, Greece; 2 Institute of Marine Biology, Biotechnology and Aquaculture, Hellenic Centre for Marine Research, 71003 Heraklion, Crete Greece

**Keywords:** *Dentex*, Soymeal, Digestive tract, Hsp70, p38 MAPK, Bcl-2, Caspase-3

## Abstract

**Background:**

Soybean is a common alternative protein source of plant origin in aquafeeds as it has a reasonably balanced amino acid profile and is widely available. This study aimed to investigate the influence of partial substitution of fish meal with soy meal on cytoprotective pathways and apoptosis in the digestive system of common dentex (*Dentex dentex*), using the activation of Hsp70, p38 MAPK, Bcl-2 and caspase-3. The experimental approach involved feeding of common dentex with three isoprotein and isoenergetic diets that contained fish meal as a protein source (FM), partial replacement of fish meal by soy meal 25% (SM25) and 40% (SM40) for 3 months.

**Results:**

The SM40 diet induced Hsp70 activation only in the middle part of intestine. On the other hand, both SM25 and SM40 diets diminished the phosphorylation of p38 MAPK in the anterior and the middle part of intestine, whereas only SM25 induced p38 MAPK phosphorylation in the stomach. Moreover, a decrease in the levels of caspase-3 activity was observed in the middle and posterior intestine, as well as in the stomach after feeding with SM25 diets. Furthermore, Bcl-2 levels were increased by SM40 in the anterior and by SM25 in the middle part of intestine.

**Conclusions:**

SM25 and SM40 diets elicited a tissue and soy concentration specific cellular and cell protective response in the different parts of the digestive tract in common dentex.

## Background

The rapid development of aquaculture combined with the increasing fishing pressure on the wild fish stocks as well as the high cost of fish meal, makes the substitution of fish meal with plant derived proteins compulsory [e.g. 1]. Various plant products have been suggested as potential agents for aquafeeds to support the sustainable production of various fish species in captivity [2]. Although many plant substitutes are used, information regarding the effect of these meals on fish health and welfare is limited [3]. Moreover, the antinutritional factors contained in plant ingredients limit their use as alternative protein sources [4]. Even though there are some techniques, such as thermal processing that decrease the presence of antinutrients, the tolerance of each fish species to these substances varies [4].

Soybean is considered the most interesting substitute of fish meal [5] due to its availability in global markets [6] and its’ reasonably balanced amino acid profile [2]. In common dentex (*Dentex dentex*), partial substitution of fish meal is possible with 25% of soy protein concentrate without affecting growth, which can be improved further by dietary taurine supplementation of 2 g kg^−1^ diet [7]. However, soybean has limited lysine and methionine content, it contains antinutritional factors and its palatability is low [4, 7, 8]. In an earlier study [7], it was found that parameters of growth, specific growth rate, feed intake, feed utilization and condition factor in common dentex fed with the SM25 diet were not affected negatively; however, the fish fed with SM40 were affected since growth, specific growth rate, feed intake, feed utilization and condition factor were decreased. Moreover, the SM40 diet resulted in increased mortality of dentex compared to the FM and SM25 diets. On the other hand, the SM content in the diet of common dentex did not affect the proximate composition of the muscle and the liver of the fish [7], although there was a tendency for a decrease in the hepatic fat content with the increase of soy in the diet. Furthermore, there have been observed some negative effects of soybean meal in the health aspect of some fish species [7, 9–11] and for this reason, its impact on fish health, welfare and the molecular level of organization needs to be examined. In marine organisms including fish, a common metric to assess the cellular response to stress is the evaluation of certain bio-indicators such as the expression of several proteins (e.g. heat shock proteins-Hsps), the enzymatic activity levels, or DNA and protein damage [12–15].

Hsps are intracellular proteins that are induced in cells in response to stress, in order to protect essential cellular functions [16]. Many studies illustrate that the induction of Hsps, including Hsp70, helps the survival of the stressed fish [17]. Although, fish is an ideal model to examine Hsps, as they can be studied at all life span of fish [17], the response of these stress proteins vary according to the tissue, the Hsp family and the stressor [18]. Evidence indicating the activation of mitogen-activated protein kinases (MAPKs) in the induction of Hsps is derived from studies on mammalian tissues [19–21] and fish tissues [22]. MAPKs act as information messenger-molecules, transferring signals from the plasmatic membrane to the inner cell resulting to immediate gene transcription and regulation [23]. Specifically, in *Sparus aurata* red blood cells, p38 MAPK mediates the induction of Hsp70 in response to thermal stress [22]. The p38 MAPK subfamily is a key signal-transducer of responses to several stresses and pro-inflammatory cytokines [24, 25]. Another critical process for the elimination of redundant, ectopic, damaged, mutated or infected cells is apoptosis. Therefore, apoptosis, a genetically controlled and evolutionarily conserved form of active cell death, plays an essential role in tissue homeostasis, [26]. The intrinsic/mitochondrial pathway, regulated by the Bcl-2 family proteins and mainly characterized by mitochondrial membrane permeabilization (MMP) [27–29] is a main distinct apoptosis signalling pathway. Caspase-3, as other caspase members (caspases-2, -6, -7, -8, -9, -10 and -14) [30], is a distinct member of the apoptosis pathway. All caspases are primarily synthesized as inactive enzymes (pro-caspases). When activated, they take part in the pro-apoptotic pathway which finally induces apoptosis under stress conditions [31].

In fish, the digestive tract is susceptible to exogenous factors such as bacteria, physical environment and diet [32]. Several studies show that different nutritional challenges, such as long starvation, or dietary lipid content, or taurine supplementation affect the induction and phosphorylation of heat shock proteins (Hsps) and members of the MAPK family respectively in farmed fishes [33–35].

Herein, the molecular indicators Hsp70, p38 MAPK, Bcl-2 and caspase-3 activity were studied in the anterior, middle and posterior intestine, as well as in the stomach in common dentex, in order to evaluate the effect provoked by the partial soybean meal substitution of fish diets on cytoprotective pathways and on apoptosis.

## Methods

### Chemicals

All biochemicals were purchased from Sigma (Darmstadt, Germany), Cell Signaling (Beverly, MA, USA) and Bio-Rad (Hercules, CA). All other chemicals were obtained from Sigma (Darmstadt, Germany), Merck (Darmstadt, Germany) and Applichem (Gatersleben, Germany) and were of analytical grade.

### Fish rearing

The feeding experiment was conducted at Institute of Marine Biology, Biotechnology and Aquaculture, Hellenic Centre for Marine Research (HCMR), Greece using juvenile common dentex, *Dentex dentex*, with an initial body weight of 39.1 ± 0.7 g. The fish originated from a common stock obtained by mesocosm hatchery technology, weaned and adapted to a compound diet (LT-Power; Perseus Specialty Food Products SA, Zevgolatio, Greece).

### Diet preparation

Three isoprotein diets (Table 1) were formulated and fed to triplicate groups of fish (9 tanks) to investigate the effect of partial substitution of fish meal (FM) by soybean meal (SM) product (HP-300) [0 (FM), 25% SM (SM25) and 40% SM (SM40)] on cytoprotective pathways and on apoptosis. All diets were prepared in the laboratory. Briefly, the dietary ingredients were thoroughly mixed in a feed mixer and moistened by the addition of 50% (w/v) water, and then converted to pellets by a mincing machine. The pellets were cut into shape manually, dried by an air-drier at 35 °C for 24 h and stored in a freezer at − 15 °C until used.

**Table 1:** Composition of experimental diets (dry basis)

	FM	SM25	SM40
Ingredients (g kg^−1^)			
Fish meal LT^a^	696.38	522.28	417.83
Soybean meal^b^	0.00	223.21	357.14
α-Starch	80.00	40.00	20.00
Vitamins and minerals^c^	20.00	20.00	20.00
Binder, Na alginate	20.00	20.00	20.00
Choline chloride	4.00	4.00	4.00
Fish oil^d^	148.89	155.16	157.15
Cellulose	30.73	15.34	3.88
Chemical composition			
Protein (% DM)	50.10	50.20	49.46
Fat (% DM)	21.50	21.46	20.57

^a^TripleNine Fish Protein, Denmark

^b^HP 300 Hamlet Protein, Denmark

^c^Roche Vitamin and Mineral mix for Aquaculture No. 5024: vitamin A: 2,500,000 Units kg^−1^; cholecalciferol: 1,000,000 Units kg^−1^; tocopherol acetate: 100 g kg^−1^; vitamin K_3_: 5 g kg^−1^; thiamine: 15 g kg^−1^; riboflavin: 15 g kg^−1^; pyridoxine: 12 g kg^−1^; cyanocobalamin: 0.25 g kg^−1^ nicotinic acid: 60 g kg^−1^; pantothenic acid 25 g kg^−1^; folic acid: 2.5 g kg^−1^; biotin: 0.5 g kg^−1^; cobalt 1 g kg^−1^; iodine 1.5 g kg^−1^; selenium: 0.05 g kg^−1^; Iron: 50 g kg^−1^; magnesium 40 g kg^−1^; copper 2 g kg^−1^; zinc 50 g kg−^1^

^d^Sardine Oil Winterisation Europe, France

### Experimental conditions

HCMR installations at Crete are Licensed Facilities for operations of breeding and experimentation use of fish (Region of Crete, General Directorate of Agricultural and Veterinary under the updated Licence No. 3989/01.03.2017). The breeding and experimental facilities were registered with the following approval codes: EL91-BIObr-03 and EL91-BIOexp-04. The experimental procedures were performed following the Greek presidential decree no. 56 and Official Journal of the Greek Government No. 106/30 April 2013 enforcing the Directive 2010-63-EU on the protection of animals used for scientific purposes.

Twenty fish were housed in each 500 l tank supplied with biologically filtered seawater (salinity 39‰) and renewed at a 200% per hour, pumped directly from 4 m beneath the surface of the sea and oxygenated to above 70% saturation by an air supply. The temperature was that of the natural seawater and ranged from 24 °C at the start of the experiment in October to 18 °C at the end of December, while the photoperiod was maintained at 13 h light/11 h dark, throughout the experimental period. Before starting the experiments, fish were acclimated for a week to the experimental conditions and fed on FM diet. Then, three tanks were randomly assigned to each diet. The fish were hand fed twice a day (at 9:00 and 17:00) to apparent satiation, over the course of 3 months.

At the end of the feeding trial, 10 of the fish from each tank (30 fish for each diet) were sacrificed by anesthesia overdose (tricaine methanesulfonate-MS222, Sigma Aldrich, St. Louis, MO, USA). For the present study, 24 different animals in total, 8 (randomly selected from the three different tanks of each diet) for each diet were used. Thereafter, the digestive tract of each fish was removed and rinsed three times in distilled water. Afterwards, the digestive tract was dissected in the following tissues: anterior, middle and posterior intestine and stomach. Immediately after, tissues were frozen in liquid nitrogen and stored at − 80 °C for further analysis of the activation of Hsp70 and phosphorylation of p38 MAPK, measurement of Bcl-2 expression levels and caspase-3 activity.

### SDS-PAGE and immunoblot analysis

Frozen tissues were homogenized in 3 ml g^−1^ of cold lysis buffer (20 mM β-glycerophosphate, 50 mM NaF, 2 mM EDTA, 20 mM Hepes) (4-(2-hydroxyethyl)-1-piperazineethanesulfonic acid), 0.2 mM Na_3_VO_4_, 10 mM benzamidine, pH 7, containing 200 μM leupeptin, 10 μΜ *trans*-epoxy succinyl-l-leucylamido-(4-guanidino)butane, 5 mM DTT (dithiothreitol), 300 μΜ phenyl methyl sulfonyl fluoride (PMSF) and 1% v/v Triton X-100), and extracted on ice for 30 min. Samples were centrifuged (10,000*g*, 10 min, 4 °C) and the supernatants were boiled with 0.33 volumes of SDS/PAGE sample buffer [330 mM Tris–HCl, 13% v/v glycerol, 133 mM DTT, 10% w/v SDS (sodium dodecyl sulfate), 0.2% w/v bromophenol blue]. Protein concentration was determined using the Bio-Rad protein assay (Bio-Rad, Hercules, CA, USA).

Equal amounts of protein (50 μg) were separated on 10% (w/v) acrylamide, 0.275% (w/v) bisacrylamide slab gels and transferred electrophoretically onto nitrocellulose membranes (0.45 μm, Schleicher and Schuell, Keene N.H. 03431, USA). Non-specific binding sites on the membranes were blocked with 5% (w/v) non-fat milk in TBST (Tris Buffered Saline-Twin 20) [20 mM Tris–HCl, pH 7.5, 137 mM NaCl, 0.1% (v/v) Tween 20] for 30 min at room temperature. Subsequently, the membranes were incubated overnight with the appropriate primary antibodies. Antibodies used were as follows: monoclonal mouse anti-heat shock protein, 70 kDa (Sigma, Darmstadt, Germany), polyclonal rabbit anti-phospho-p38 MAP kinase (Thr180–Tyr182) (Cell Signaling, Beverly, MA, USA), polyclonal rabbit anti-p38 MAP kinase (Cell Signaling, Beverly, MA, USA) and polyclonal rabbit anti-Bcl-2 (Abcam, Cambridge, MA, USA). After washing in TBST (3 × 5 min), the blots were incubated with horseradish peroxidase-linked secondary antibodies, polyclonal goat anti-mouse immunoglobulins and polyclonal goat anti-rabbit immunoglobulins (DAKO, High Wycombe, Buckinghamshire, UK), washed again in TBST (3 × 5 min), and the bands were detected by enhanced chemiluminescence (Cell Signaling, Beverly, MA, USA) with exposure to Fuji Medical X-ray films. Blot bands were quantified by laser scanning densitometry (GelPro Analyzer Software, Media Cybernetics). Equal protein loading was verified by staining identical samples with Ponceau S staining solution (0.1% w/v Ponceau S in 5% v/v acetic acid).

### Colorimetric determination of caspase-3 activity

Caspase-3 activity was determinate in tissues of digestive system according to Calbiochem’s protocol (Cat No. 235400). Briefly, the tissues were homogenized in lysis buffer which includes the appropriate inhibitors (lysis buffer pH 7.4: HEPES 50 mM, NaCl 100 mM, CHAPS 0.1%, DTT 1 mM, EDTA 0.1 mM; inhibitors: leupeptin, NP40, E64, PMSF). Protein concentration was estimated with Bio-Rad protein assay (Bio-Rad, Hercules, CA, USA). Caspase-3 inhibitor I (500 nM) was added to the relevant wells with assay buffer, protein extract and caspase-3 substrate. Control wells (blank) contain only assay buffer and caspase-3 substrate. The absorbance was measured at 405 nm in a microtiter plate reader after 2, 4 and 6 h of incubation, respectively. Then, the data was illustrated at a graph (OD versus time) and only the data from the early portion of the curve were taken into consideration in due to calculate the slope, in view of the reduce of the substrate to sub-saturating levels. In this experiment, the data after the 2 h measurement was used.

### Statistics

Changes in the levels of Hsp70, p38 MAPK phosphorylation, Bcl-2 and caspase-3 activity under different diet conditions were tested for significance at the 5% level by using Kruskal–Wallis and Mann–Whitney tests. Values are presented as mean ± SD.

## Results

**Fig. 1:**
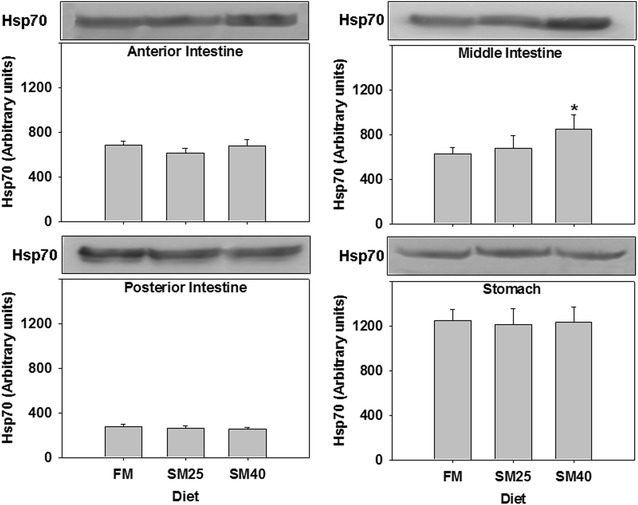
Induction of Hsp70 in the anterior, middle, posterior intestine and stomach of common dentex (*Dentex dentex*) under the FM, SM25 and SM40 diet treatment. Representative protein bands are shown on the top of the panel. Western blots are representative of at least three independent experiments with overlapping results and data represent mean ± SD for at least three independent experiments; n = 8 preparations from different animals. *p < 0.05 compared to FM; ^#^ *p* < 0.05 compared to SM25

SM40 diet resulted in elevated Hsp70 in the middle part of the intestine in common dentex compared to the control FM group (*p* < 0.05). On the other hand, no changes (*p* > 0.05) were observed in the other examined tissues, including the stomach, the anterior and posterior intestine of fish fed with the SM25 and SM40 compared to the FM diet (Fig. 1).

**Fig. 2:**
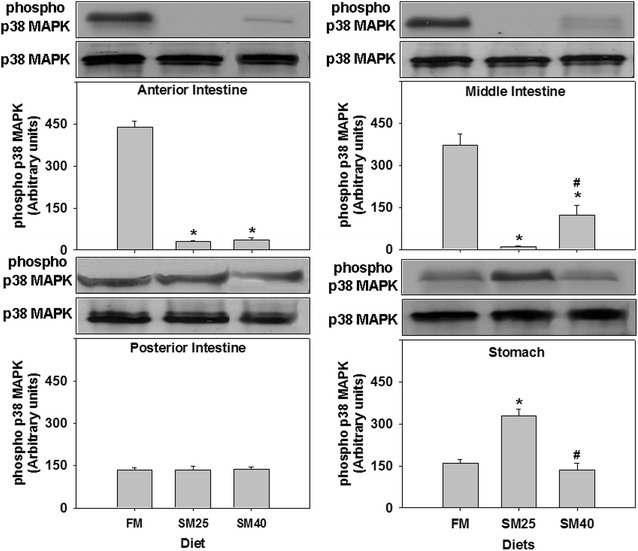
Phosphorylation of p38 MAPK in the anterior, middle, posterior intestine and stomach of common dentex (*D. dentex*) under the FM, SM25 and SM40 diet treatment. Representative protein bands are shown on the top of the panel. Western blots are representative of at least three independent experiments with overlapping results and data represent mean ± SD for at least three independent experiments; n = 8 preparations from different animals. *p < 0.05 compared to FM; ^#^ *p* < 0.05 compared to SM25

Phosphorylated levels of p38 MAPK were diminished in the anterior and middle intestine in fish fed the SM diets (SM25 and SM40) compared with fish fed the FM diet (*p* < 0.05) (Fig. 2). In the middle intestine, however, SM40 fish had significantly higher phosphorylated p38 MAPK levels compared to SM25 (*p* < 0.05). The SM diets (SM25 and SM40) did not alter the phosphorylation of p38 MAPK in the posterior intestine (*p* > 0.05). On the other hand, p38 MAPK exhibited elevated phosphorylation in the stomach only under the SM25 diet compared with FM group (*p* < 0.05) whereas, SM40 diet had no effect on p38 MAPK phosphorylation compared with SM25 group (*p* > 0.05) (Fig. 2).

**Fig. 3:**
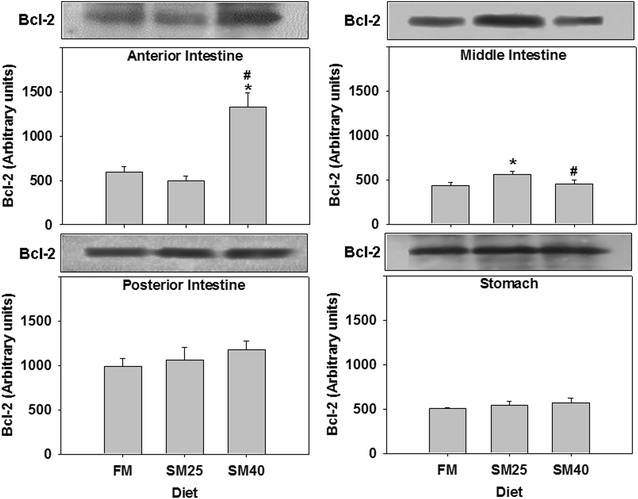
Bcl-2 levels in the anterior, middle, posterior intestine and stomach of common dentex (*D. dentex*) under the FM, SM25 and SM40 diet treatment. Representative protein bands are shown on the top of the panel. Western blots are representative of at least three independent experiments with overlapping results and data represent mean ± SD for at least three independent experiments; n = 8 preparations from different animals. *p < 0.05 compared to FM; ^#^ *p* < 0.05 compared to SM25

The effect of the soy based diets on the anti-apoptotic protein Bcl-2 is shown in Fig. 3. In the anterior part of the intestine, only SM40 diet resulted in elevated Bcl-2 levels compared with both FM and SM25 diets (*p* < 0.05). On the other hand, an increase in Bcl-2 levels was observed in the middle part of the intestine in fish under SM25 regime (*p* < 0.05). The diets had no effect on Bcl-2 in the posterior part of the intestine and the stomach (*p* > 0.05) (Fig. 3).

**Fig. 4:**
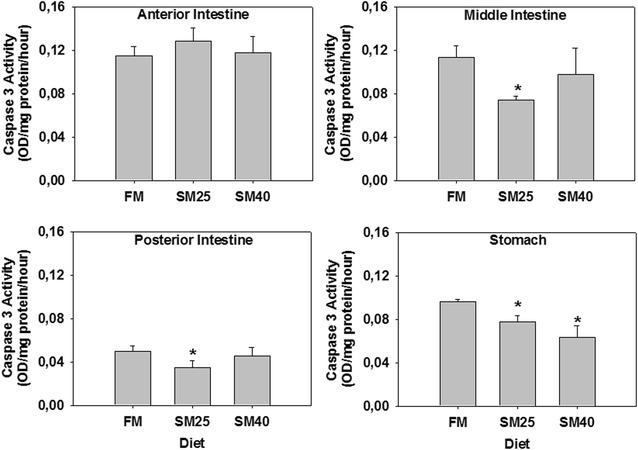
Caspase-3 activity in the anterior, middle, posterior intestine and stomach of common dentex (*D. dentex*) under the FM, SM25 and SM40 diet treatment. Values represent mean ± SD for at least three independent experiments; n = 8 preparations from different animals. *p < 0.05 compared to FM

Contrary to the anterior intestine, where no changes in the activity levels of caspase-3 were observed (*p* > 0.05), SM25 diet decreased caspase-3 activity in the middle, posterior intestine and stomach (*p* < 0.05), while SM40 diet decreased caspase-3 activity only in the stomach (*p* < 0.05) (Fig. 4). The most profound changes were observed in the middle intestine and stomach.

## Discussion

Information regarding the effect of diet composition or feeding strategies on certain molecular indicators of the cellular response or the apoptosis pathway in fish is scarce. The present results showed generally a differential and distinct tissue-specific induction of Hsp70, phosphorylated p38 MAPK and Bcl-2, as well as caspase-3 activity, under the effect of fish meal substitution by different soy protein concentrates (SM25 and SM40), in the four examined tissues of the digestive tract (anterior, middle, posterior intestine and stomach) in common dentex.

Recently, several studies investigated the expression of Hsps in relation to various nutritional challenges. Food deprivation induces and thus leads to elevated Hsp70 and Hsp90 levels in larval gilthead seabream (*Sparus aurata*) and rainbow trout (*Oncorhynchus mykiss*) [36]. Furthermore, in Indian carp, *Labeo rohita*, fingerlings starvation increases the Hsp70 levels [37]. Similarly, in the European sea bass (*Dicentrarchus labrax*), starvation and re-feeding affected the activation of Hsp70 and Hsp90 in a tissue specific pattern [33]. Specifically, starvation led to increased Hsp70 levels in liver and red muscle, while starvation and re-feeding caused the decrease of Hsp70 levels in the same tissues albeit the increase of Hsp90 levels, which seemed to remain unaffected under starvation. On the other hand, levels of Hsp90 increased in liver and red muscle when fish were starved and re-fed. In the white muscle, both starvation and starvation and re-feeding led to the decrease of Hsp90 levels, while both Hsps remained unaffected under both feeding regimes [33]. Also, the feeding regime of different dietary lipid contents affects the induction of hepatic Hsps in meagre (*Argyrosomus regius*) [34]. Specifically, the 17 and 21%-lipid diets resulted in elevated hepatic Hsp70 levels compared with fish of 13%-lipid diet, while Hsp90 levels were increased only in fish fed the 17%-lipid diet [34]. In the juvenile white seabass (*Atractoscion nobilis*) at ambient temperature, Hsp70 and Hsp60 responses in muscle and liver were positively correlated with the increase of dietary lipid levels [38]. In addition, the more the feeding rate increases, the higher seems to be the increase of Hsps levels in the liver of larval white sturgeon (*Acipenser transmontanus*) [39]. Intestinal Hsp70 levels are also influenced after replacing fish meal with plant proteins in Atlantic cod (*Gadus morhua*) [10].

The present study showed that Hsp70 expression profile varied between tissues and different soy protein concentrates in common dentex. Specifically, only SM40 caused a significant increase in Hsp70 levels in the middle part of the intestine. In accordance with these results, elevated (but not statistically significant) levels of Hsp70 is found in the middle part of the intestine in Atlantic cod after a substitution of fish meal in a regression design up to 440 g kg^−1^ with plant ingredients [8]. In another study on Atlantic cod, an increase of Hsp70 is found only in fish in which fish meal protein was replaced with a 100% plant protein diet and no changes in Hsp70 levels in the 25–75% plant protein diets [10]. In European sea bass, a 60% replacement of soy protein concentrate in the fish diet causes a decrease in Hsp70 protein levels in the liver, while Hsp90 expression levels were elevated both in the liver and anterior intestine [35]. Thus, the above data together with the results of the present study indicate a differential Hsp response, pointing out a different mode of action for these molecules in the cellular defence process, depending on the fish species, the examined tissue and the nutritional state.

Several studies have reported that similarly to Hsp expression, MAPK activation differs not only between tissues of the same species, but also between different species and stimuli [40–42]. Hsp induction and MAPK phosphorylation levels were found to be different depending on different factors including the time course of heat stress [43] or the examined tissue of fish [e.g. 33–35, 44].

Contrary to the large amount of data concerning Hsps in fish, there is very little available information concerning MAPK activation by different dietary effects in fish [33–35]. Most studies in fish relate the induction of members of the MAPK family with stimuli such as thermal stress, hypertonic or hypotonic shock, increased salinity and the effect of chemical agents [45–49]. Although there are numerous studies, which have showed the induction of Hsps via MAPK signalling pathways [19–22], the present results show no correlation between the Hsp70 levels and the activation levels of p38 MAPK in the examined tissues. Specifically, in the present study, although both SM25 and SM40 diminished the phosphorylation of p38 MAPK in the anterior and middle part of the intestine in common dentex, elevated Hsp70 levels were only found in the middle intestine of the SM40 fish. Moreover, SM25 resulted in elevated phosphorylated levels of p38 MAPK in the stomach, whereas Hsp70 levels were not affected (Table 2).

**Table 2:**
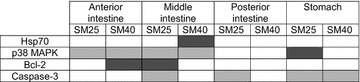
Changes in the levels of the examined proteins between SM25 and SM40 compared to control FM treatment (dark grey indicates increase, light grey indicates decrease and white indicates no change compared to 4F-0S)

In the common dentex, the effect of the soymeal diet on the phosphorylation of p38 MAPK was appeared to be tissue specific in the digestive track. Moreover, in the middle part of the intestine and in the stomach, the effect was different in the fish fed the different SM diets. The dietary effects of soy protein on the protein members of MAPK family appear to be species and dose specific. In the sea bass, 60% replacement of soy protein concentrate in fish diet increases p38 MAPK phosphorylation in the liver and the posterior intestine, as well the phosphorylation of ERKs in the anterior intestine [35], although diminished phosphorylation of p38 MAPK was observed under SM25 and SM40 diet in the common dentex. In the liver of meagre, the 17 and 21%-lipid diets activate the phosphorylation of p38-MAPK, while the phosphorylation ratio of both extracellular signal-regulated kinases (ERKs) and c-Jun N-terminal kinases (JNKs) were mostly observed at 17%-lipid diet [34].

To the knowledge of the authors, there is no available data connecting the MAPK pathway to the apoptotic pathway in fish. Information connecting these different pathways derives only from mammalian tissues [50–56]. The molecular mechanisms associating the above mentioned pathways are not fully understood in fish, since available information on these proteins in amphibians and fish mostly concern morphogenesis [57–60]. Probably different kinds of external factors (e.g. changes in oxygen availability or in osmotic environment, or even the diet) and physiological processes, such as intestinal epithelium renewal, are closely related to the apoptotic pathway [e.g. 61]. In this sense, in the present study, the decrease of apoptotic events in the digestive tract of fish fed the SM diets could possibly indicate a decrease in the rhythm of physical epithelium renewal. Specifically, the anti-apoptotic Bcl-2 levels in the anterior and middle intestine were increased under the effect of the SM diets in common dentex, while levels of the apoptotic caspase-3 activity decreased in fish fed mainly the SM25 diet comparing to the control fish of FM diet in the middle, posterior intestine and stomach. Thus, these results show a positive effect of the SM diets in the anti-apoptotic pathway and therefore a decrease in apoptosis despite the positively correlated increased mortality incidents parallel to the soy meal substitution increase [7] in the present study. However, the epithelium renewal rhythm is out of the scope of the present investigation and remains to be studied. On the other hand though, many researchers found that the substitution of fish meal with plant protein (e.g. soy protein) did not affect negatively the health of cultivated fish species [62–64]. Contrary to our results, in Atlantic salmon (*Salmo salar*), caspase-3 levels were increased in the intestine epithelium after feeding with soybean meal [9]. In addition, intestine epithelium folds were also found to be more atrophic and short compared to the control [9]. Thus, probably a differential apoptotic response is activated in different species and under different stimuli. Moreover, it is worth mentioning that although, all the examined tissues in the present study possess a key role in the process of food utilization, the involvement of the cellular and apoptotic responses in digestion, is far more complex and requires focused studies in order to be elucidated since limited research has focused on the physiological cell turnover and apoptosis in the fish intestines under dietary condition.

## Conclusions

The partial substitution of fish meal with SM25 and SM40 soymeal in the diet of the common dentex stimulates a differential cellular response including the HSR (Heat Shock Response), the MAPK and the apoptotic pathway. Specifically, the changes observed in Hsp70 expression, p38 MAPK activation, Bcl-2 levels and caspase-3 activity reveal a response that might be tissue distinct in the digestive track and could be related to the effect elicited by different contents of soy protein diets. However, a more holistic approach on the biology of farmed fish fed with different diets such as the soy based diets is needed in order that molecular, behavioural and physiological responses could be combined for the evaluation of the maintenance of fish adequate health and stock.
